# An Analysis of Anki Usage and Strategy of First-Year Medical Students in a Structure and Function Course

**DOI:** 10.7759/cureus.23530

**Published:** 2022-03-27

**Authors:** David M Harris, Michael Chiang

**Affiliations:** 1 Medical Education, University of Central Florida College of Medicine, Orlando, USA; 2 Academic Affairs, University of Central Florida College of Medicine, Orlando, USA

**Keywords:** anatomy and physiology, flashcards, medical education, spaced repetition, retrieval practice

## Abstract

It is well known that undergraduate medical education demands learners to master large amounts of material. To help cope with learning, medical students have resorted to e-learning resources that include video lectures, visual mnemonics, and flashcard systems. The purpose of the current study was to determine the usage of Anki (Damien Elmes, https://apps.ankiweb.net/) among first-year medical students in an integrated first-year module consisting of anatomy and physiology. Additionally, this study aimed to elucidate how students used Anki in conjunction with their lectures. Sixty first-year medical students were recruited in a twelve-week longitudinal study consisting of three surveys (Weeks 1, 7, and 13) about usage of Anki and their perceptions of how to use it with formal lecture. About 70% of participants utilized Anki in the course and the usage increased significantly from Week 1 to Weeks 7 and 13. There was variation to how many days a week students used Anki. Survey data shows that students value formal lectures and prefer to supplement their studies with Anki. A large proportion of first-year medical students use Anki to supplement their studies. Faculty should consider ways to incorporate Anki into their teaching to facilitate student learning through active recall and spaced repetition.

## Introduction

Undergraduate medical education demands learners to master large amounts of material at an alarming pace. Additionally, students are learning other skills such as clinical reasoning and physical exam skills that can add to cognitive load. To help cope with learning pace, medical students have resorted to e-learning resources that include video lectures, visual mnemonics, and flashcard systems. Although Kim et al. found that most of the faculty in their study had positive perceptions of e-learning resources, far less incorporated any into their teaching. The barriers to utilizing e-learning resources were lack of resources relevant to lecture, lack of time to use it during lecture and lack of awareness of these resources [1]. Faculty need to understand how pervasive these e-learning resources are and how students are using them, to consider how to help students navigate them or incorporate features into the curriculum. It is known that large numbers of students use these tools to study for the United States Medical Licensing Exam (USMLE) Step 1. However, how students use these resources earlier in the medical school curriculum is less known[2-4].

Many e-learning resources are based on established learning principles. For example, Anki (Damien Elmes, https://apps.ankiweb.net/) is a software program that allows users to make digital flashcards, which can consist of pictures, videos, or audio [5]. Its success is based on the scientific principles of active recall and spaced repetition that involves showing new and more difficult flashcards more frequently than easier and less difficult flashcards to promote the “spacing effect” [6]. In 2015, Deng et al. showed that students had a one-point increase on their licensing exams for every 1,700 unique Anki flashcards used which has circulated on the online student forums [7]. There have been other studies reporting the positive effect of question banks on licensing examination performance as well [2,3,8]. The majority of these studies focused on resources students used outside of the formal curriculum. It has been acknowledged that spaced repetition should be included in future medical school education, yet there is limited literature to how [9,10].

Few studies ask students how they use e-learning resources. By knowing how and why students use e-learning resources, faculty could integrate these educational tools into formal curricula to enhance learning. The purpose of the current study was to determine Anki usage among first-year medical students in an integrated first-year module consisting of anatomy and physiology. Usage consisted of number of cards, as well as the number of days a week a student utilized Anki. Additionally, this study aimed to elucidate how students used Anki in conjunction with their lectures. 

## Materials and methods

Context and participants

The University of Central Florida’s (UCF's) Institutional Review Board determined this study to be exempt. The current study took place during the Structure and Function Module of the UCF College of Medicine Medical Doctor (MD) Program. The module occurred in the first year of the program and ran from October to February. The module was 16 weeks long and included the disciplines of gross anatomy, microanatomy, embryology, medical imaging, and physiology. The pedagogies utilized in this module included lectures, gross anatomy dissections, small group case-based learning activities and high-fidelity patient simulations. Sixty of the 120 medical students (50%) at the UCF College of Medicine were recruited to participate in this study.

Anki usage survey and analysis

Participants were provided a Qualtrics survey at Weeks 1, 7, and 13 of the Structure and Function module. The survey asked students if they used Anki, the numbers of cards per week, and the number of days spent reviewing Anki flashcards. Participants were compensated for each survey of the study and were the same throughout the study weeks. A non-parametric Friedman test with a post-hoc Wilcoxon signed-rank test was used to determine whether there was a significant difference in Anki usage within the sample across the three time points.

Study habit survey and analysis

Participants were provided a Qualtrics survey at Week 13 that asked questions about study habits, how students perceived lecture and Anki, and how they utilized Anki. This 31-question survey used a seven-point scale with 1 = strongly disagree, 4 = neutral, and 7 = strongly agree. After converting students’ responses to a numerical value, a T-statistic 99% confidence interval was generated for the sample mean for each response to determine if any of the responses were significant. A significant response is any generated confidence interval that does not include the theoretical mean of 4 (a value where students would neither agree nor disagree with the statement asked).

## Results

**Figure 1:**
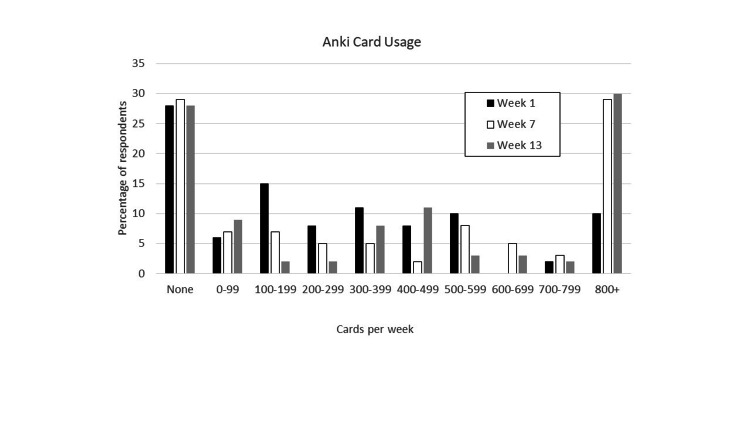
Number of Anki cards reviewed per week

A survey question asked the number of Anki cards that were used per week to determine how pervasive Anki usage was among the first-year medical students. Figure 1 shows that over the course of the study there was about the same percentage of students (28-29%) that did not utilize the Anki cards. The other 70% or so utilized various numbers of cards per week in Week 1 from less than 100 to over 800 cards. There was a significant increase in the numbers of cards used between weeks 1 and 7 (P = .001). There was no significant increase in the numbers of cards used between Weeks 7 and 13. These data show that most of the first-year medical students surveyed utilized Anki and that the number of cards used increased as the course progressed from Week 1 to Week 7. 

**Figure 2:**
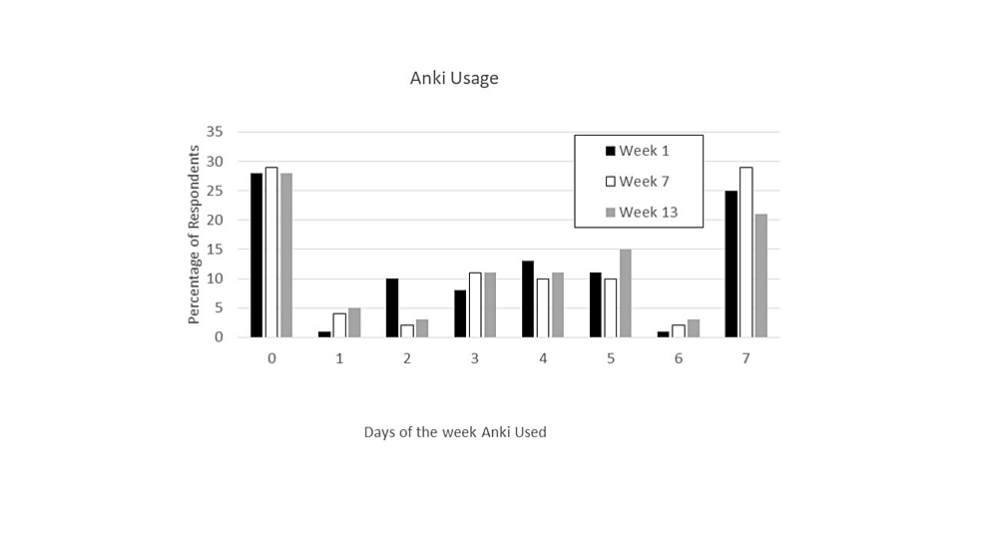
Number of days Anki was used

To further gauge the usage of Anki, a second survey question asked how many days per week the first-year medical students used Anki. Figure 2 shows that the same number of students did not use Anki at all similar to the first question. The other 70% students used Anki for different amounts of days per week with the largest percentage using Anki seven days a week. There were no significant differences in the days per week Anki was used across the course. These data suggest that how students employ Anki cards across the week is variable despite the increasing numbers of cards generated over time.

A seven-point Likert-like survey was deployed at Week 13 to elucidate student perceptions on the usage of Anki. The results are shown in Table 1. Twenty-one out of 31 questions were related to Anki or the use of external resources. Questions 3 to 6 provide data on the relationship of external resources and lecture. These data show that students still perceive value to lecture. However, students perceived that they need to supplement their learning with outside resources as the classroom lectures were not sufficient. This was supported by Question 9 in which students disagreed that Anki was a waste of time.

**Table 1:** Survey questions and analyses Likert scale values: 1 = strongly disagree, 4 = neutral, 7 = strongly agree.  A significant response is any generated confidence interval that does not include the theoretical mean of 4 (a value where students would neither agree nor disagree with the statement asked).

Survey Question	Mean	SD	Lower Limit	Upper Limit	Significant?
1. I find the classroom lecture a good use of my time.	5.10	1.64	4.48	5.72	Yes
2. I find the classroom resources sufficient for my studying.	4.02	1.76	3.35	4.69	No
3. I feel comfortable just using classroom resources for my studying plan.	3.26	1.76	2.59	3.93	Yes
4. I need to use outside resources to supplement classroom lectures.	5.66	1.29	5.17	6.15	Yes
5. I think outside resources could replace all of our classroom lectures.	3.56	1.89	2.84	4.28	No
6. My time is better spent using outside resources than looking at lectures.	3.42	1.80	2.74	4.10	No
7. I find lectures a waste of time.	2.82	1.68	2.18	3.46	Yes
8. I do not like using Anki	3.88	2.31	3.00	4.76	No
9. I think Anki is a waste of time.	2.94	2.05	2.16	3.72	Yes
10. I feel the material is relevant and useful for my future.	5.50	1.08	5.09	5.91	Yes
11. I feel the material is relevant and useful for my Board Examination.	5.66	1.03	5.27	6.05	Yes
12. I think Anki should be included in the curriculum.	3.72	2.13	2.91	4.53	No
13. I keep up with the material as it is presented in class.	5.48	1.51	4.91	6.05	Yes
14. I study a regular amount from day to day.	5.22	1.53	4.64	5.80	Yes
15. I usually cram my studying at the last moment.	3.12	1.81	2.43	3.81	Yes
16. I can retain the information I learn long-term.	4.62	1.25	4.15	5.09	Yes
17. I am quick to forget the information I learn.	3.78	1.38	3.26	4.30	No
18. I use Anki primarily for long-term retention of material.	3.78	1.97	3.03	4.53	No
19. I use Anki primarily for quick cramming of material.	2.94	1.79	2.27	3.61	Yes
20. I use Anki to discover gaps within my learning.	4.40	1.85	3.70	5.10	No
21. My studying time is decreased when using Anki.	3.60	2.07	2.82	4.38	No
22. I feel my mastery of material is increased using Anki.	4.44	1.99	3.69	5.19	No
23. I feel that Anki helps me gauge what material is expected of me.	3.78	1.96	3.04	4.52	No
24. I think Anki causes me to focus too much on small details.	4.16	1.76	3.49	4.83	No
25. Using Anki increases my stress level.	4.06	1.97	3.31	4.81	No
26. Using Anki decreases my stress level.	3.64	1.84	2.94	4.34	No
27. I prefer to use my own Anki cards.	2.32	1.74	1.66	2.98	Yes
28. I prefer to use Anki cards passed down from other people.	5.18	1.85	4.48	5.88	Yes
29. I prefer to watch the lecture first and to do Anki afterwards.	5.22	1.93	4.49	5.95	Yes
30. I prefer to do Anki and then watch the lecture afterwards.	1.92	1.28	1.43	2.41	Yes
31. I only use Anki for studying but do not watch lecture.	1.84	1.41	1.31	2.37	Yes

Questions 18 to 31 provide perceptions of how students used Anki in relationship to their studies. For Question 19, students disagreed with the statement that Anki was used primarily to quickly cram material. Questions 27 and 28 indicate that students prefer to use Anki cards that were previously developed rather than create their own. Questions 29 and 30 show that students prefer to watch a lecture before doing Anki cards. These data support the thought that students like to supplement formal lectures with Anki cards as opposed to replace.

## Discussion

The main finding from this study is that about 70% of the 50% of surveyed first-year medical students used Anki to help them study for the Structure and Function module at UCF College of Medicine. The second finding is that the number of Anki cards and daily use of Anki cards varied among users. Study habit and perception data about external resources suggested that students perceived the need to supplement their learning with external resources, that students preferred to use already made cards as opposed to creating their own, and students preferred to do Anki after watching a lecture.

In this study, about 70% of the student participants used Anki to supplement their studies in our first-year course. There were about 30% that did not use Anki and this remained steady throughout the course suggesting that those students most likely did not adopt this method of study. A recent study provided curriculum specific Anki cards related to anatomy and just over 50% of students used the cards and responded to the surveys [11]. It is important to note that the cards in that study were content related to the class and not focused on board preparation, which is similar in our study. A study by Banos et al. provided class access to a question bank for the initial 18 months of the curriculum without directives and found variable use in their systems-based modules ranging from 22% usage to 94% [2]. Other studies have shown the use of external resources such as First Aid and UWorld’s Qbank to be over 99% although that was focused on Step 1 preparation and closer to the licensing exam as opposed to early in the curriculum [8]. Consistent with other studies, our study suggests that Anki flashcards are not used by a portion of students. Further studies are needed to understand why, or why not, a student chooses to use this tool in the early curriculum.

There have been less studies focusing on the motivation of students to use Anki. Surveys have shown that faculty have concerns regarding quality, support and incentives with the increasing prevalence of online learning [12]. Faculty concerns include the loss of the faculty role and the future of faculty [13]. Our data supports the use of lecture which is consistent with studies that show that the large majority of medical students watch or stream lectures by institutional faculty [14-16]. Our data suggests that students utilize Anki to supplement their studies as opposed to replace formal instruction. Importantly, students do not feel that formal lectures are adequate for studying (Questions 3 and 4). Our data suggests that students are using Anki to solidify their study efforts as they prefer to use it after lecture. (Questions 29-31) The use of Anki flashcards use active recall as students use them as a question bank, which is the most popular use of electronic online resources [17]. Overall, the survey data suggests that students are using Anki to supplement their classroom instruction because they find it relevant to their future and board exam preparation.

A limitation of the current study is that the data was collected in one medical school and involved a portion of the first-year medical student class. Since it only included one curriculum, we do not know if it is generalizable to the other schools that may have different curricula. There could also be sample bias as students interested in Anki could be more willing to participate. However, with 30% of the participants not adopting Anki usage and most likely never adopting through the study suggest that bias may be limited. This is also a self-reported survey, which may not actually reflect card usage.

## Conclusions

The data suggest that large numbers of medical students use Anki for studying anatomy and physiology early in the curriculum. They also suggest that student use Anki to supplement the instruction provided by the faculty as opposed to replacing it. Since Anki is based on active recall and spaced repetition, faculty should consider how best to implement a similar strategy in their classroom to help a certain portions of student studying. 
